# Development of Viral-Vectored Vaccines and Virus Replicon Particle-Based Neutralisation Assay against Mayaro Virus

**DOI:** 10.3390/ijms23084105

**Published:** 2022-04-07

**Authors:** Young Chan Kim, Arlen-Celina Lücke, César López-Camacho, Beate Mareike Kümmerer, Arturo Reyes-Sandoval

**Affiliations:** 1 The Jenner Institute, Nuffield Department of Medicine, University of Oxford, The Henry Wellcome Building for Molecular Physiology, Roosevelt Drive, Oxford OX3 7BN, UK; cesar.lopez-camacho@ndm.ox.ac.uk (C.L.-C.); arturo.reyes@ndm.ox.ac.uk (A.R.-S.); 2 Institute of Virology, Medical Faculty, University of Bonn, 53127 Bonn, Germany; arlen.luecke@uni-bonn.de; 3 German Centre for Infection Research (DZIF), Partner Site Bonn-Cologne, 53127 Bonn, Germany; 4 Instituto Politécnico Nacional (IPN), Av. Luis Enrique Erro s/n., Unidad Adolfo López Mateos, Mexico City 07738, Mexico

**Keywords:** Mayaro virus, Chikungunya virus, viral-vectored, adenovirus, MVA, vaccine, enzyme-linked immunosorbent assay (ELISA), immunogenicity, neutralisation assay

## Abstract

Mayaro virus (MAYV) is an emerging alphavirus causing acute febrile illness associated with chronic polyarthralgia. Although MAYV is currently restricted to tropical regions in South America around the Amazon basin, it has the potential to spread globally by *Aedes* species mosquitoes. In addition, there are currently no specific therapeutics or licenced vaccines against MAYV infection. We have previously shown that an adenovirus based Mayaro vaccine (ChAdOx1 May) was able to provide full protection against MAYV challenge in vaccinated A129 mice and induced high neutralising antibody titres. In this study, we have constructed a replication deficient simian adenovirus (ChAdOx2) and a Modified Ankara Virus (MVA) based vaccine expressing the MAYV structural cassette (sMAYV) similar to ChAdOx1 May, and characterised recombinant MAYV E2 glycoprotein expressed in a mammalian system for immune monitoring. We demonstrate that ChAdOx2 May was able to induce high antibody titres similar to ChAdOx1 May, and MVA May was shown to be an effective boosting strategy following prime vaccination with ChAdOx1 or ChAdOx2 May. In order to measure MAYV neutralising ability, we have developed a virus replicon particle-based neutralisation assay which effectively detected neutralising antibodies against MAYV. In summary, our study indicates the potential for further clinical development of the viral vectored MAYV vaccines against MAYV infections.

## 1. Introduction

Mayaro virus (MAYV) is a mosquito-borne alphavirus that belongs to the family *Togaviridae* and causes Mayaro fever (MAYF) characterised by flu-like symptoms including fever, myalgia, arthralgia, and skin rash [1,2,3]. Although MAYV is currently restricted to Central and South America, causing small sporadic outbreaks [2,3,4,5], it has the potential to pose a risk in countries where *Aedes* spp. mosquitoes are present, affecting millions of people worldwide, similar to the Chikungunya virus (CHIKV) [1,2,4,6,7,8].

Despite this, there are no licensed vaccines available to date, and only a few MAYV vaccines were shown to be immunogenic in mouse models, including an inactivated MAYV vaccine [9], a live-attenuated virus vaccine based on a recombinant virus for which the MAYV subgenomic promotor was replaced by an internal ribosome entry site (IRES) [10], and a DNA-based vaccine or a non-replicating human adenovirus encoding the MAYV structural polyprotein [11,12].

MAYV is an enveloped, positive-strand RNA virus belonging to the genus *Alphavirus* within the family *Togaviridae* [13]. As described for other alphaviruses, the genome is nearly 12 kilobases in length and encodes four non-structural (ns) and five structural (s) proteins [13]. The non-structural proteins, nsP1, nsP2, nsP3, and nsP4 are required for RNA replication. The structural proteins capsid (C), E3, E2, 6K, and E1 are encoded by a subgenomic RNA resulting in a polyprotein that is cleaved by the capsid auto-proteinase as well as cellular signalases [13,14]. The two envelope glycoproteins, E1 and E2, form heterodimers and compose the surface of the virions [15,16,17]. While E1 mediates fusion, E2 interacts with the host receptor and represents the major target for neutralizing antibodies [17,18,19,20,21,22,23].

We have previously reported that ChAdOx1 encoding MAYV structural proteins (ChAdOx1 May) elicited rapid and robust immunity with high titres of neutralizing antibodies against MAYV, able to protect A129 mice from a lethal challenge and reducing viremia to undetectable levels [24]. It was also shown that vaccination with ChAdOx1 May offers some degree of cross-protection against a lethal CHIKV challenge [24]. Our viral vectored chikungunya vaccine (ChAdOx1 Chik) has recently completed a Phase 1 trial, and it was shown to be safe and highly neutralising against multiple lineages of CHIKV after a single dose [24,25,26]. Despite this, ChAdOx1 Chik offers limited cross-protection against a lethal MAYV challenge [24].

ChAdOx2 is a novel recombinant replication deficient chimpanzee adenovirus, and the first clinical use of ChAdOx2 as a vaccine vector demonstrated better tolerability and less reactogenicity compared to its predecessor ChAdOx1 vector vaccine [27,28]. Therefore, in this study, we developed ChAdOx2 and Modified Ankara Virus (MVA) based vaccines expressing the structural cassette of MAYV (sMAYV). Furthermore, we expressed and characterised recombinant MAYV E2 glycoprotein expressed in a mammalian system for immune monitoring. In order to determine the ability to neutralise MAYV, we also developed a virus replicon particle (VRP) based neutralisation assay which was shown to effectively detect neutralising antibodies against MAYV. Using these developed MAYV vaccines and tools, we could show that our viral-vectored vaccines elicit functional neutralising antibodies against MAYV and induce cross-neutralising antibodies against CHIKV. In summary, our study indicates the potential for further clinical development of the viral vectored MAYV vaccines against MAYV infections.

## 2. Results

### 2.1. Construction of ChAdOx2 and MVA May

**Figure 1:**
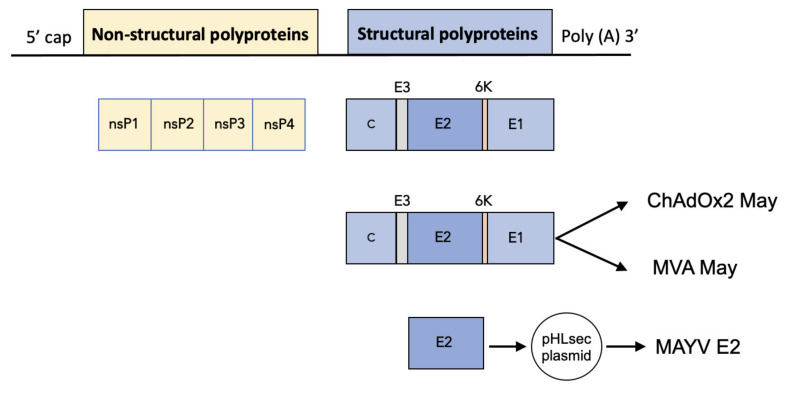
Mayaro virus (MAYV) genome organization and design of viral-vectored May vaccines and recombinant MAYV E2 glycoprotein. On (**top**): Lines represent non-translated regions and boxes represent translated regions, whereas the yellow boxes represent the non-structural proteins and the blue boxes the structural proteins. (**Below**): arrows indicate translated regions that were cloned into the indicated vaccine vectors or the mammalian expression plasmid pHLsec.

We constructed a replication deficient simian adenovirus (ChAdOx2) and a Modified Ankara Virus (MVA) based vaccine expressing the MAYV structural polyproteins (capsid, E3, E2, 6K, and E1) to produce ChAdOx2 May and MVA May, respectively (Figure 1). To produce and characterise recombinant MAYV E2 with a C-terminal His tag, the MAYV E2 gene was cloned into the pHLsec expression vector resulting in pHLsec-MAYV E2 (Figure 1).

### 2.2. Characterisation of MAYV E2

**Figure 2:**
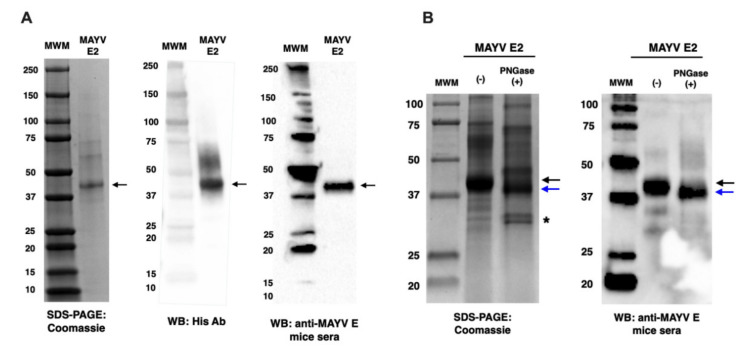
Characterisation of MAYV E2 glycoprotein. (**A**) Purified MAYV E2 glycoprotein was analysed by SDS-PAGE following Coomassie-staining gel or WB analysis using an anti-His tag antibody or anti-MAYV E reactive mice sera. The arrow indicates the band specific to MAYV E2. (**B**) N-glycosylation of MAYV E2 glycoprotein was analysed by SDS-PAGE Coomassie-stained gel and WB with anti-MAYV E reactive mice sera. Samples were either loaded without PNGase F treatment (-) or with PNGase F treatment (+). The black and blue arrows indicate the band specific to MAYV E2 without PNGase F treatment (-) or with PNGase F treatment (+), respectively. The symbol (*) indicates the band specific to PNGase F enzyme.

To produce and characterise recombinant MAYV E2 glycoprotein, HEK293T cells were transfected with pHLsec-MAYV E2, and the secreted MAYV E2 was purified by nickel column chromatography. The purified MAYV E2 was analysed by SDS-PAGE Coomassie staining and Western blot (WB). Specific bands of ~40 kDa corresponding to MAYV E2 lacking the C-terminal transmembrane (TM) domain were visualised after Coomassie staining or WB analysis using an anti-His tag antibody or anti-MAYV E reactive mice sera, respectively (Figure 2A). It was predicted that MAYV E2 is N-glycosylated at N261 using the GlycoEP software [29]. In order to check for N-glycosylation of MAYV E2 glycoprotein produced in HEK293T cells, MAYV E2 was treated with a PNGase F to remove all N-linked glycans and analysed by SDS-PAGE Coomassie staining and Western Blot (WB) using an anti-MAYV E reactive mouse sera (Figure 2B). PNGase F-treated MAYV E2 glycoprotein showed a reduced molecular mass both for the Coomassie stained SDS-PAGE gel and the WB, which suggests that MAYV E2 was efficiently N-glycosylated.

### 2.3. Immunogenicity of ChAdOx2 and MVA May Vaccines

**Figure 3:**
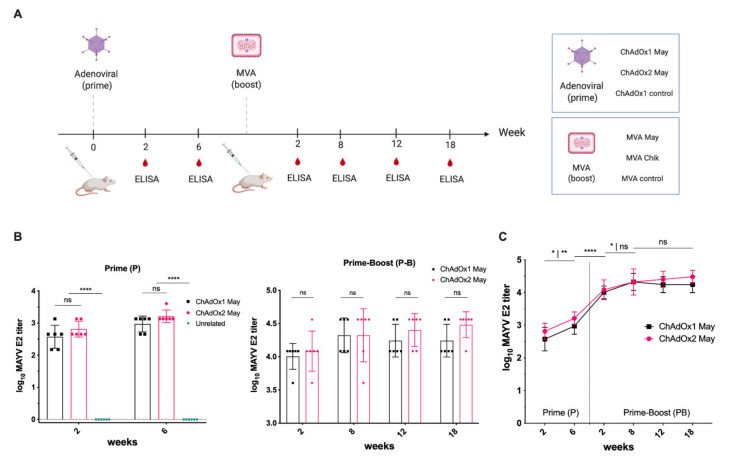
Description of vaccination regimen and immunogenicity elicited by ChAdOx1/ChAdOx2 -MVA MAYV vaccines. (**A**) A group of BALB/c mice (*n* = 6) were immunised with ChAdOx1 May or ChAdOx2 May. Unrelated ChAdOx1 DENV NS1 vaccine was used as a control. All mice were boosted with MVA encoding the same antigens. Serum samples were collected at 2 or 6 weeks after prime immunisation and 2, 8, 12, and 18 weeks after P-B immunisations. (**B**) Humoral IgG responses elicited by the vaccines after prime (**left**) or prime-boost (**right**) were quantified by ELISA in plates coated with MAYV E2. The reciprocal log MAYV E2 ELISA titers were calculated for all groups. (**C**) MAYV E2 specific antibody body titers over time are shown as kinetics. Coloured lines represent the mean with SD (*n* = 6 per group) and error bars. *p* values were determined by one-way ANOVA and Tukey’s multiple comparisons test. *p* > 0.05 (ns), *p* < 0.05 (*), *p* < 0.01 (**), and *p* < 0.0001 (****).

To assess the specific immunogenicity of ChAdOx2 May vaccine and to compare it with the ChAdOx1 May vaccine, we immunised groups of BALB/c mice (*n* = 6) with a single and unadjuvanted dose of ChAdOx2 May or ChAdOx1 May at 1 × 10^8^ infectious units (IU) per animal (Figure 3A). Further, a group receiving unrelated ChAdOx1 dengue virus (DENV) NS1 was included as a control. The same groups were then boosted with MVA vaccines encoding the same antigens. Specific IgG antibody responses against MAYV E2 antigen were measured by ELISA at 2 weeks and 4 weeks after prime (P) immunisation and 2, 8, 12, and 18 weeks after prime-boost (P-B) immunisations. Two weeks post-prime immunisation, the mean MAYV E2-specific antibody titers elicited by ChAdOx1 May and ChAdOx2 May were 2.57 and 2.81, respectively, and there was no statistical significance between the two groups (Figure 3B). No specific IgG antibody binding to MAYV E2 was detected in the ChAdOx1 DENV NS1 group. By week 6, the anti-MAYV E2 titers for both ChAdOx1 May and ChAdOx2 May vaccinated animals increased to 2.97 and 3.21, with no statistical difference between the two groups (Figure 3B,C). MVA vectored vaccines were previously shown to be good as an adjuvant in heterologous prime-boost regimen while they were not very effective when given as a prime vaccine [30]. Therefore, to assess the immunogenicity of MVA May vaccines as a boost vaccine, the same groups were boosted with MVA vaccines encoding the same antigens at a dose of 1 × 10^6^ plaque-forming units (PFU) per mouse. Two weeks after the boost, the anti-MAYV E2 titers for both groups increased significantly (*p* < 0.0001) to reach 4.01 and 4.10, respectively (Figure 3B,C). This increased further by 8 weeks after the boost, and the levels stayed similar at 4.24 and 4.48, respectively, even at 18 weeks post-boost vaccination. This suggests that similar antibody titers are elicited by both ChAdOx1 and ChAdOx2 May vaccines when given as a prime, and the MVA boosting is an effective strategy.

### 2.4. Immunogenicity of ChAdOx2 and MVA May Vaccines

**Figure 4:**
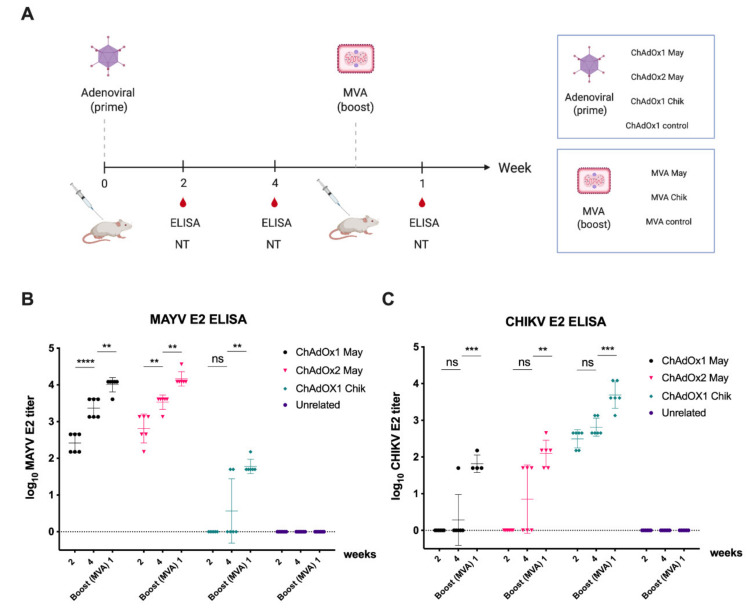
Description of vaccination regimen and humoral responses elicited by adenoviral-MVA based MAYV and CHIKV vaccines. (**A**) A group of BALB/c mice (*n* = 6) were immunised with one of adenoviral vaccines (May or Chik) or unrelated ChAdOx1 DENV NS1 vaccine. All mice were boosted with MVA encoding the same antigens. Serum samples were collected at 2 or 4 weeks after prime immunisations and 1 weeks after P-B immunisations for immunogenicity (ELISA) and neutralisation assays (NT). (**B**,**C**) Humoral IgG responses elicited by the vaccines were quantified by ELISA in plates coated with MAYV E2 or CHIKV E2. Coloured lines represent the mean with SD (*n* = 6 per group) and error bars. *p* values were determined by one-way ANOVA and Tukey’s multiple comparisons test. *p* > 0.05 (ns), *p* < 0.01 (**), *p* < 0.001 (***), and *p* < 0.0001 (****).

We have previously reported immunogenicity, neutralisation, and in vivo protection of ChAdOx1 Chik and ChAdOx1 May in mice [24,25,31]. Here, we sought to assess the cross-reactive immunogenicity and neutralisation of viral vectored MAYV vaccines. To assess the degree of cross-reactivity against CHIKV and vice versa, groups of mice (*n* = 6) were immunised with either ChAdOx1 May or ChAdOx2 May and ChAdOx1 Chik. Further, an unrelated ChAdOx1 DENV NS1 group was included as a control (Figure 4A). The same groups were then boosted with MVA vaccines expressing the same antigens to assess the extent of reactivity and cross-reactivity after the MVA boost.

IgG antibody responses against MAYV and CHIKV E2 were measured by ELISA at 2 weeks and 4 weeks after prime immunisation and 1 week after prime-boost immunisation (Figure 4B,C). No IgG antibody responses against MAYV E2 or CHIKV E2 were detected for the unrelated control group (ChAdOx 1 DENV NS1) (Figure 4B,C). Two weeks post-immunisation, the mean MAYV E2-specific antibody titers elicited by ChAdOx1 May and ChAdOx2 May were 2.41 and 2.81, respectively (Figure 4B). No IgG antibody binding to MAYV E2 was detected in the ChAdOx1 Chik group. By week 4, the anti-MAYV E2 titers for both ChAdOx1 May and ChAdOx2 May vaccinated animals increased to 3.37 and 3.53, respectively, while 2 of 6 animals vaccinated with ChAdOx1 Chik showed cross-reactivity towards MAYV E2 antibodies. By 1 week after MVA boost, the anti-MAYV E2 titers for both ChAdOx1 May and ChAdOx2 May groups increased to 4.01 and 4.16, respectively, while ChAdOx1 Chik vaccinated animals showed cross-reactivity towards MAYV E2 with the mean antibody titer of 1.78 (Figure 4B). Similarly, IgG antibody responses against CHIKV E2 were measured for all vaccinated groups (Figure 4C). At 2 weeks post-prime, the mean CHIKV E2-specific antibody titer elicited by ChAdOx1 Chik was 2.49, and no IgG antibody binding to CHIKV E2 was seen in ChAdOx1 and ChAdOx2 May groups. By week 4, the anti-CHIKV E2 titers for the ChAdOx1 Chik group had increased to 2.81, while some animals vaccinated with ChAdOx1 May (1 out of 6) or ChAdOx2 May (3 out of 6) showed cross-reactivity towards CHIKV E2 antigen. By 1 week after MVA boost, the anti-CHIKV E2 titers for ChAdOx1 Chik increased to 3.69, while ChAdOx1 May and ChAdOx2 May vaccinated animals showed cross-reactivity towards CHIKV E2 antigen with the mean antibody titer of 1.82 and 2.10, respectively. In summary, these results suggest that cross-reactive antibodies can be elicited by both ChAdOx1 and ChAdOx2 May vaccines against CHIKV E2 and vice versa, which can be increased by the MVA boosting.

### 2.5. Establishment of MAYV VRP-Based Neutralisation Assay

**Figure 5:**
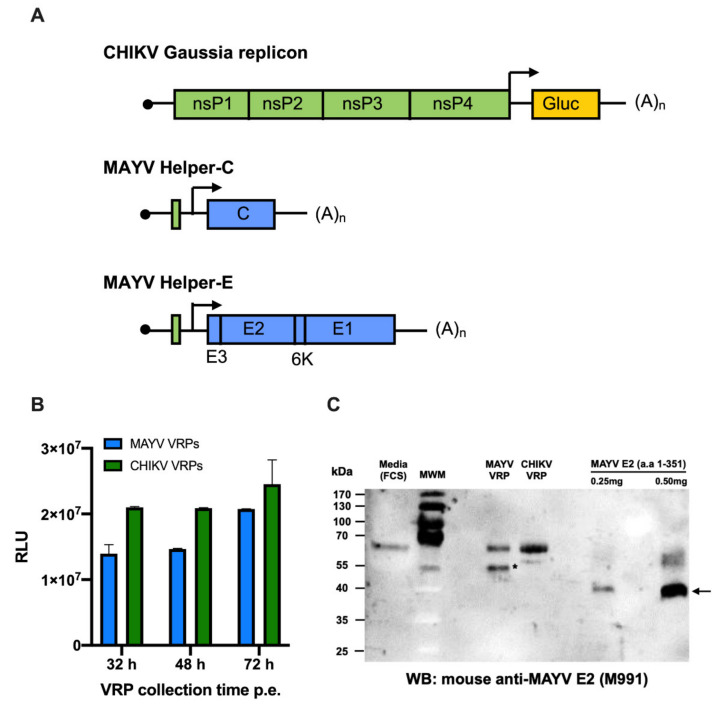
MAYV VRP system. (**A**) Schematic representation of the CHIKV replicon expressing Gluc marker and the MAYV helper-C and MAYV helper-E RNAs. Lines represent non-translated regions and boxes represent translated regions. Green boxes indicate non-structural proteins of CHIKV, and blue boxes indicate MAYV structural proteins. The Gluc reporter is represented as a yellow box. In the helper constructs 5′ terminal nucleotides of the CHIKV nsP1 gene, important for RNA replication, were retained. Arrows indicate the position of the subgenomic promoter and the solid black circle at the 5′ end of each RNA represent the CAP structure and (A)_n_ indicates the poly (**A**) tail. (**B**) Optimum of MAYV-VRP production. Same volumes of the supernatants collected at different time points after co-electroporation of replicon RNA and helper RNAs were used to infect fresh BHK cells. At 24 h post infection, Gaussia luciferase released into the supernatant was measured. Data represent the mean with range of duplicate infection experiments. (**C**) MAYV VRP were analysed by WB using a mouse anti-MAYV E2 monoclonal antibody (M991). The symbol (*) indicates the band specific to MAYV E2 in MAYV VRPs and the arrow indicates the recombinant MAYV E2 glycoprotein without C-terminal membrane domain (positive control) produced in this study. Media (MEM + FCS) was used a negative control. MWM: protein marker.

We have previously reported a virus replicon particle (VRP)-based CHIKV neutralisation assay using Gaussia luciferase (Gluc) as read out [32]. To determine the neutralisation ability of our ChAdOx1 May and ChAdOx2 May vaccines, in this work we developed a MAYV neutralisation assay based on chimeric MAYV virus replicon particles (VRPs). The MAYV VRPs were produced by co-transfecting BHK-21 cells with a CHIKV replicon expressing Gaussia luciferase (Gluc) [32] and two helper plasmids expressing the MAYV capsid protein C (pMayHelper-C) or the envelope proteins (pMayHelper-E), respectively (Figure 5A). Electroporated cells were incubated at 32 °C and supernatants were harvested at 32 h, 48 h, and 72 h post electroporation (p.e.) to assess the efficiency of VRP production produced at different times p.e. via infection of fresh BHK cells. For comparison, the analogous experiment was performed for the production of CHIKV-Gluc VRPs. Compared to the CHIKV-VRPs, production of the chimeric MAYV VRPs was only slightly reduced at all time points (less than half) with the highest yield at 72 h p.e. (Figure 5B). Hence, for optimized MAYV-VRP production, the coelectroporated cells were incubated at 32 °C and harvested at 72 h p.e.

In order to further test the secretion of MAYV VRPs into the supernatant, WB using a mouse anti-MAYV E2 monoclonal antibody (M991) was carried out using the supernatant containing MAYV VRP or CHIKV VRP (Figure 5B). MAYV VRP showed the expected sized band, ~55 kDa, corresponding to the MAYV E2, demonstrating the MAYV VRP is secreted efficiently and binds specifically to mouse anti-MAYV E2 monoclonal antibodies (M991). No similar sized band was seen in CHIKV VRP, indicating that it did not cross-react with the anti-MAYV E2 mAb used in this study. Purified recombinant MAYV E2 glycoprotein lacking the C-terminal membrane domain produced in this study was used as a positive control and it showed specific bands of ~40 kDa corresponding to MAYV E2. Negative control (MEM + FCS) showed non-specific band ~65 kDa, and similar bands were also seen in the supernatant of MAYV VRP and CHIKV VRP containing MEM + FCS.

### 2.6. MAYV Neutralisation—Neutralising Ability in Vaccinated Mice Sera

**Figure 6:**
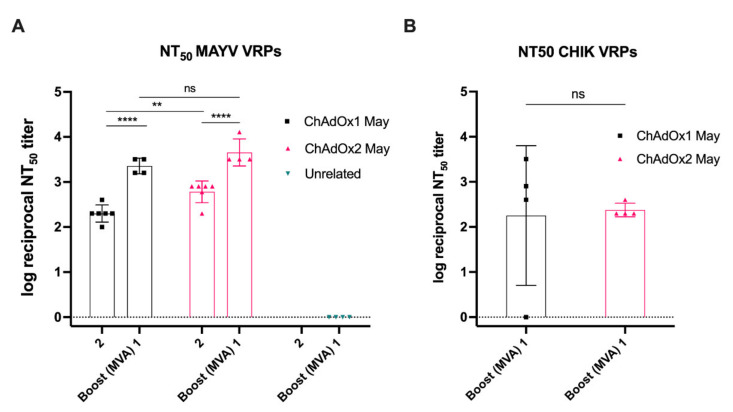
MAYV and CHIKV neutralisation assays. (**A**) MAYV NT_50_ titers for BALB/c mice serum samples 2 weeks post-prime immunisation with ChAdOx1 or ChAdOx2 May or unrelated ChAdOx1 NS vaccine. NT_50_ titers 1 week post MVA boost are shown. (**B**) CHIKV NT_50_ titers for ChAdOx1/MVA May and ChAdOx2/MVA May are shown. Coloured lines represent the mean with SD and error bars. *p* values in (**A**) were determined by one-way ANOVA and Tukey’s multiple comparisons test. *p* > 0.05 (ns), *p* < 0.01 (**), and *p* < 0.0001 (****). *p* value in (**B**) was determined by the Mann-Whitney U test. *p* > 0.05 (ns).

To assess the MAYV neutralising ability in vitro, we used our MAYV VRP neutralisation assay to determine NT_50_ values for BALB/c mice sera obtained after an early prime time-point (2 weeks post-prime ChAdOx1 or ChAdOx2 May) and after prime-boost vaccination (1 week post-boost MVA). An unrelated group (ChAdOx1 DENV NS1-MVA NS1) was included as negative control. At 2 weeks post-vaccination, sera from the ChAdOx1 May and ChAdOx2 May vaccinated animals showed high neutralisation activity against MAYV VRPs with reciprocal log NT_50_ titers of 2.30 and 2.78, respectively (Figure 6A). Following an MVA boost, the NT_50_ titers for ChAdOx1 May-MVA May and ChAdOx2 May-MVA May further increased to 3.35 and 3.66, respectively (Figure 6A). ChAdOx1 DENV NS1-MVA DENV NS1 vaccinated animals had no detectable NT_50_ titers. These results indicate that both ChAdOx1 and ChAdOx2 May can induce MAYV neutralising antibodies. Next, a CHIKV VRP neutralisation assay was performed to determine the cross-neutralising ability of viral-vectored MAYV vaccines against CHIKV. The ChAdOx1 May-MVA May group showed detectable NT_50_ titers in 3 out of 4 animals, with a mean NT_50_ titer of 2.25, while neutralising antibodies were detected in all animals vaccinated with ChAdOx2 May-MVA May, yielding a higher NT_50_ titer of 2.38. These results suggest that viral-vectored MAYV vaccines can, upon a heterologous prime-boost regimen, induce high titers of cross-neutralising antibodies against CHIKV. Taken together, our results confirm that our viral-vectored vaccines elicit functional neutralising antibodies against MAYV-infective particles tested by an in vitro model and induce cross-neutralising antibodies against CHIKV-infective particles.

## 3. Discussion

In this study, we have developed MAYV vaccines based on viral-vector platforms such as ChAdOx2 and MVA vectors. ChAdOx2 is a novel recombinant replication deficient chimpanzee adenovirus and the first clinical use of ChAdOx2 as a vaccine vector demonstrated better tolerability and less reactogenicity at the 5 × 10^10^ viral particles (vp) dose than its predecessor ChAdOx1 vector vaccine [27,28]. MVA vectored vaccines were previously shown to be good as a boost following prime immunisation with adenoviral vectored vaccines [30]. Therefore, in this study, we first performed immunization with ChAdOx2 expressing the MAYV structural cassette sMAYV (ChAdOx2 May), followed by a boost using the corresponding MVA MAYV vaccine (MVA May). To allow monitoring of anti-MAYV E2 antibody levels, recombinant MAYV E2 glycoprotein was produced in mammalian cells and verified by SDS-PAGE and WB using an anti-His Ab and ChAdOx1 May vaccinated mice serum. N-glycosylation analysis of MAYV E2 demonstrated that this antigen was efficiently N-glycosylated in HEK293 cells. The immunogenicity of ChAdOx2 May and MVA May vaccines were tested by assessing humoral immune response in BALB/c mice by ELISA using our recombinant MAYV E2. Our data suggest that ChAdOx2 May induces similar high anti-MAYV E2 titres than ChAdOx1 May after 2 weeks post-prime immunisation and the anti-MAYV E2 titres for both groups increased significantly upon boost with MVA May. It was also shown that cross-reactive antibodies could be elicited by the ChAdOx2 May vaccine similar to ChAdOx1 May against CHIKV. Furthermore, it was also shown that the antibody titers were significantly increased for both groups by boosting with a corresponding MVA May vaccine.

To allow the determination of neutralising MAYV antibodies, we developed a neutralisation assay based on chimeric MAYV virus replicon particles (VRPs) for which a CHIKV replicon expressing Gluc was packaged in trans by the MAYV structural proteins [32]. Compared to the homologous CHIKV VRP system (CHIKV replicon and CHIKV helper constructs), production of the MAYV VRPs was slightly delayed, but reached similar levels at 72 h post electroporation. For safety reasons, a split helper system was used to provide the structural proteins [33]. Using this strategy, the chance of recombination possibly resulting in infectious full-length virus is negligible [21]. This is especially an important aspect with regard to biosafety issues, as MAYV is classified as BSL3 agent. Besides the advantage that the MAYV VRP assay does not need to be performed in a BSL3 facility, as is the case for the classical MAYV plaque reduction neutralisation assay, it also is less time consuming. The assay can be performed in a 96-well high throughput format, and the use of secreted Gluc as reporter protein allows the assay to easily be evaluated from the supernatant.

The established MAYV VRP assay was used to assess MAYV antibody neutralisation activity in vitro and determine NT_50_ values which showed that ChAdOx2 May was able to induce significantly higher NT_50_ titers than ChAdOx1 May at 2 weeks post-immunisation. However, the NT_50_ titers for both groups became similar after boosting with MVA May with no statistical difference between two groups. As ChAdOx1 May was previously demonstrated to offer a full protection against MAYV challenge in mice with high neutralising PRNT antibody titers, ChAdOx2 May is also likely to induce high PRNT antibody titers and afford full protection in a MAYV challenge model [24]. Given better tolerability and less reactogenicity of ChAdOx2 than ChAdOx1 in clinical testing, combined with data from this study showing similar or greater NT_50_ titers, ChAdOx2 May should be further explored as a MAYV vaccine candidate [27,28]. Our study has also shown that viral-vectored MAYV vaccines upon heterologous prime-boost regimen can induce high titers of cross-neutralising antibodies against CHIKV. A live attenuated MAYV vaccine (MAYV/IRES) has recently been evaluated which provided protection against MAYV challenge in both immunocompetent and immunocompromised (A129) mouse models [34]. PRNT_50_ titers, determined following MAYV/IRES vaccination in mice, showed that it was able to induce specific humoral response against MAYV but not against CHIKV [34]. A recent study by Powers et al. reported a MAYV vaccine based on non-replicating human adenovirus V (AdV) which elicited high neutralising antibodies that protected both wild-type and immunocompromised mice against MAYV challenge upon homologous prime-boost vaccination with the AdV [12]. It was also shown that high MAYV neutralisation antibody titers confer cross-protection against two other alphaviruses such as CHIKV and Una virus (UNAV). Cross protection obtained against CHIKV is in agreement with results from this study. Our previous study did not induce significant titers of cross-neutralising antibody upon single immunisation of ChAdOx1 Chik or ChAdOx1 May, despite conferring significant cross-protection against heterologous disease [24]. Therefore, it will be interesting to assess if our heterologous (ChAdOx–MVA) regimen is able induce high levels of cross-neutralising antibodies against other arthritogenic alphaviruses such as the Ross River virus (RVV), O’nyong nyong virus (ONNV), and Barmah Forest virus (BFV) [24].

Taken together, our results confirm that our viral-vectored vaccines elicit functional neutralising antibodies against MAYV-infective particles, tested by an in vitro model as well as inducing cross-neutralising antibodies against CHIKV-infective particles. Given a better tolerability and less reactogenicity of ChAdOx2 than ChAdOx1, ChAdOx2 May should be tested further in clinical studies with heterologous MVA boost to increase the cross-reactivity against other arthritogenic alphaviruses and to assess the potential of our viral-vectored vaccines to be developed as a multi-alphavirus targeting vaccine.

## 4. Materials and Methods

### 4.1. Transgene Design

The structural MAYV (sMAYV) cassette (C, E3, E2, 6K, and E1) derived from various MAYV lineages was codon optimized as previously described [24]. To improve initiation of translation, a Kozak consensus sequence was included before the 5′ end of the transgene and it included the required enzymatic restriction sites [24]. A synthetic gene cassette was produced by GeneArt^®^ (Fisher Scientific, Regensburg, Germany) and was named sMAYV [24].

### 4.2. Viral-Vectored Vaccine Production

The plasmid containing the sMAYV cassette (C, E3, E2, 6K, and E1) was digested with the restriction enzymes KpnI and NotI to allow in-frame cloning of the transgene between the CMV promoter and the PolyA sequence region contained in our shuttle and expression vector (pMono). The recombinant DNA plasmid was expanded and purified from *E. coli* using the Qiagen MIDI-prep kit. Resulting plasmids were verified by restriction analysis and 5′ and 3′ flanking sequencing. To generate the ChAdOx2 vaccine, the shuttle plasmid containing attL region sequences was recombined with those attR regions contained in the destination vector (ChAdOx2) using an in vitro Gateway reaction (LR Clonase II system, Invitrogen^TM^, Carlsbad, CA, USA). Successfully recombined ChAdOx2 May (also known as ChAdOx2 sMAYV) was verified by DNA sequencing using flanking primers (forward promoter primer and Poly-(A) reverse primer). Standard cell biology and virology techniques were performed to generate the non-replicative adenoviral vectors as described previously [28]. The vectored vaccine was purified and sterile filtered to generate a vaccine at concentration of 1.3 × 10^10^ infectious units (IU) per mL. To generate MVA based vaccines, the plasmid encoding sMAYV was digested with KpnI and XhoI to allow in-frame ligation between the P7.5 promoter and the TKR locus, contained in the MVA entry plasmid. Ligated DNA plasmid was expanded in *E. coli* and a midiprep kit (Qiagen, Germantown, MD, USA) was used for plasmid purifications. The resulting plasmid was verified by restriction analysis and 5′ and 3′ flanking sequencing, and co-transfected to produce MVA May at concentration of 1.1 × 10^10^ PFU per mL using the methodology as previously described [35].

### 4.3. Design and Production of the ChAdOx1 Chik and ChAdOx1 May

ChAdOx1 Chik and ChAdOx1 May vaccines were designed and produced as previously reported [24,25,31]. The immunogenicity and efficacy profiles of ChAdOx1 Chik and ChAdOx1 May in mice had been recently demonstrated [24,25,31].

### 4.4. Animals

Female inbred BALB/c (H-2d), (6–8 weeks) were used for the assessment of immunogenicity (*n* = 6 mice per group). Mice were purchased from Envigo RMS Inc. (Bicester, UK). No randomisation was used in this work. All animals and procedures were used in accordance with the terms of the UK Home Office Animals Act Project License.

### 4.5. Vaccination

Viral vectored vaccines were thawed on ice. All vaccines were diluted in endotoxin-free PBS. ChAdOx1 or ChAdOx2 vaccines were administered at 1 × 10^8^ infectious units (IU) per mouse. For boosting, MVA vaccines were administered at a dose of 1 × 10^6^ plaque-forming units (PFU) per mouse. Animals were anesthetized using isoflurane and then injected with 25 µL of vaccine intramuscularly in each leg.

### 4.6. Production of Recombinant MAYV E2 

For expression and purification of the MAYV E2, the codon-optimized gene of MAYV E2 without TM domain (a.a. 1–351) was cloned into the pHLsec vector [36]. The pHLsec MAYV E2 plasmid (500 µg) was transfected in HEK-293T cells using polyethyleneimine (PEI) in roller bottles (surface area of 2125 cm^2^) under standard cell culture conditions. Five days after transfection cells were discarded and media was filtered through 0.22 µM disposable filters. The secreted protein was purified from the supernatant by Ni Sepharose affinity chromatography (HisTRAP^TM^, GE Healthcare, Chicago, IL, USA), using the Äkta Start chromatography system and eluted with Imidazole 500 mM. Finally, the eluted protein was dialysed using a Slide-A-LyzerTM cassette against 1X PBS. CHIKV E2 protein was produced and purified as previously described [25].

### 4.7. SDS-PAGE and Western Blot Analysis 

To characterise the purified MAYV E2 from this study, SDS-PAGE Coomassie staining and Western blot was carried out. Purified MAYV E2 was added to Laemmli sample buffer containing 20 mM β-mercaptoethanol. Samples were boiled for 5 min and loaded on a Mini-PROTEAN TGX protein gel (reducing condition) with a protein marker (Bio-Rad, Precision Plus Protein^TM^ WesternC^TM^ standard). Proteins were transferred onto nitrocellulose membranes (Bio-Rad Trans-Blot^®^ TurboT^M^). The membranes were blocked with 5% skim milk in 0.1% PBST for 1 h and then incubated with either 1:1000 anti-His antibody (mouse 6x-His Tag Antibody, MA1-21315, ThermoFisher Scientific, Waltham, MA, USA) or 1:500 anti-MAYV E reactive mice sera. After washing with PBST, membranes were incubated for 1 h with an appropriate secondary antibody using an HRP-conjugated goat anti-mouse IgG (Bio-Rad Cat. 170-6516). The membranes were washed and incubated with chemiluminescent substrate (Clarity^TM^ Western ECL Blotting Substrates, BIO-RAD, Watford, UK) prior to detecting the signal by the chemiluminescent Western blot imaging system (Image Lab, Bio-Rad, Watford, UK). As MAYV E2 protein was produced in HEK-293T cells, N-glycosylation of MAYV E2 was assessed by treating it with PNGase F to remove all N-linked glycans before analysing it on SDS-PAGE Coomassie staining and Western blot using an anti-MAYV E reactive mice serum (1:500).

### 4.8. Enzyme-Linked Immunosorbent Assay (ELISA)

Specific antibody binding to MAYV E2 or CHIKV E2 was measured by an IgG enzyme linked immunosorbent assay (ELISA) as previously described [24,25]. Briefly, mice sera were diluted in Nunc Maxisorp Immuno ELISA plates coated with the MAYV E2 or CHIKV E2 diluted in PBS to a final concentration of 2 µg/mL and incubated at room temperature (RT) overnight. Plates were washed 6 times with PBS/0.05% Tween (PBS/T) and blocked with 300 µL with Pierce^TM^ protein-free (PBS) blocking buffer (Thermo Fisher Scientific, Waltham, MA, USA) for 2 h at RT. Mouse serum was added and serially diluted 3-fold down in PBS/T with 50 µL per well as final volume and incubated for 2 h at RT. Following washing 6 times with PBS/T, bound antibodies were detected after a 1 h incubation with 50 µL of alkaline phosphatase-conjugated antibodies specific for whole mouse IgG (A3562-5ML, Sigma Aldrich, St. Louis, MO, USA). Development was achieved using 100 µL of 4-nitrophenylphosphate diluted in diethanolamine buffer and the absorbance values at OD_405_ were measured and analysed using a CLARIOstar instrument (BMG Labtech, Aylesbury, UK). Serum antibody endpoint titers were defined by an absorbance value three standard deviations greater than the average OD_405_ of the control.

### 4.9. Establishment of MAYV VRP-Based Neutralisation Assay 

In order to measure MAYV neutralising antibody titres, a new virus replicon particle-based neutralisation assay against MAYV was developed based on a previously described method for CHIKV [32]. To this end, the E3-E2-6K-E1 cassette in pChikHelper-E or the C gene in pChikHelper-C were, respectively, exchanged against the corresponding codon optimized sequences of MAYV by molecular cloning using primers shown in Table 1. RNA was in vitro transcribed from the resulting pMayHelper-E and pMayHelper-C plasmids after linearization with *Not*I using the mMESSAGE mMACHINE SP6 Kit (Invitrogen^TM^) and coelectroporated with CHIKV Gluc replicon RNA into BHK-J cells [32]. Electroporated cells were kept at 32 °C for 72 h before chimeric VRPs were harvested from the supernatant, clarified, and purified via a sucrose cushion as described before [32]. For the concentrated chimeric MAYV-VRPs, viral RNA copies/mL were determined via CHIKV real-time PCR [32].

### 4.10. CHIKV VRP-Based Neutralisation Assay

CHIKV neutralising antibody titres were determined as described previously using CHIK VRPs expressing Gaussia luciferase (Gluc) [32]. Briefly, 2 × 10^4^ BHK-21 cells were seeded in 96-well plates per well. The next day, VRPs (MOI of 5, calculated based on VRP RNA copies/mL) were preincubated with 2-fold serial dilutions of heat inactivated serum samples for 1 h at 37 °C before the mixtures were added to the 96-well plates. After incubation for 1 h at 37 °C, the inocula were removed, cells were washed with PBS, and medium was added. Readout of secreted Gaussia was performed at 24 h post infection using a Renilla luciferase assay system (Promega, Southhampton, UK). Sera-neutralisation-capacity was determined by measuring Gluc activity and relating it to Gluc readout after VRP application without serum. Results are presented as 50% neutralisation (NT_50_) titres.

## Figures and Tables

**Table 1:** List of primers used to exchange E3-E2-6K-E1 cassette in pChikHelper-E or the C gene in pChikHelper-C against the corresponding codon optimized sequences of MAYV.

Sp6Helper F	GCTGACAGCGCCTTTTTGAA
Sp6Helper R	AGGGGTTATTCGGCCCTTG
001F	TAAGAGACACACTGTACATAGCAA
002R	TGTAGCGCTGATTAGTGTTTAGATACTTG
003F	CACTAATCAGCTACAATGGATTTTCTGCCAACACAGG
004R	ACAGTGTGTCTCTTACCATTCCTCGGTGCCCTC
005F	TAAGTATGAAGGTATATGTGTCCCCTA
006R	GGTGGCCTAGGTAGCTGATTAG
007F	GCTACCTAGGCCACCATGGCTGCTCCTACAGTGACAGC
008R	ATACCTTCATACTTATCTTCTCAGGGTGATACAGGTCA

## Data Availability

Not applicable.
